# The new clinical application of bilateral-contralateral cervix clamp in postpartum hemorrhage: a retrospective cohort study

**DOI:** 10.1186/s12884-020-03518-2

**Published:** 2021-01-13

**Authors:** Qianwen Zhang, Tao Li, Yu Xu, Yayi Hu

**Affiliations:** 1 Department of Obstetrics and Gynecology, West China Second University Hospital, Sichuan University, Chengdu, Sichuan China; 2 Key Laboratory of Birth Defects and Related Diseases of Women and Children, Sichuan University, Ministry of Education, Chengdu, Sichuan China

**Keywords:** Postpartum hemorrhage, Uterine atony, Uterotonic, Cervix clamp

## Abstract

**Background:**

To assess the efficacy and safety of bilateral-contralateral cervix clamp firstly applied in postpartum hemorrhage caused by uterine tony of lower segment.

**Methods:**

Totally 47 pregnant women with postpartum hemorrhage secondary to lower uterine segment atony in vaginal delivery or after caesarean delivery were included from March 1, 2020 to May 31, 2020. According to patient’s informed consent, 22 women accepted cervical clamp to treat and 25 only used uterotonics in control group.

Then hemostatic efficacy and safety of bilateral-contralateral cervix clamp were assessed by retrospective analysis.

**Results:**

It was found that mean blood loss in clamp group was much less during vaginal delivery (656.2±72.79 g vs 811.8±86.07 g, *p* = 0.001) or after caesarean delivery (42.8±6.60 g vs 126.3±86.97 g, *p* = 0.007), and incidence of uterotonic repeated usage (81.8% vs 36, 18.2% vs 64%, *p* = 0.001) or side effect (18.2% vs 48.0%, *p* = 0.031) appeared less than control group, but there was no statistical differences on hospital stay (4.1±1.57 days vs 3.8±1.61 days, *p* = 0.535), hemoglobin (119±4.10 g vs 121.4±4.19 g, *p* = 0.058), blood transfusion (9.1% vs 12%,*p* = 0.746), surgical procedures (4.5% vs 4.0%, *p* = 0.93), also no clamp complications occurred.

**Conclusions:**

The bilateral-contralateral cervix clamp was effective and safe, this new technique could be a complementary treatment for postpartum hemorrhage.

## Background

Postpartum hemorrhage (PPH) is commonly defined as a blood loss within 24 h more than 500 ml at vaginal delivery or 1000 ml at cesarean delivery, it was mainly secondary to uterine atony, including atony of lower uterine segment [1, 2]. PPH has always been the leading cause of maternal death globally, almost resulted in 34% of 275,000 maternal deaths worldwide in 2015, and as high as 17.62% in Chinese maternal deaths according to the National Maternal and Child Health Annual Report of China in 2018 [3–5]. The World Health Organization (WHO) updated recommendation for preventing PPH is 10 IU of oxytocin for all births, and uterotonics such as carbetocin, ergometrine and misoprostol could help uterine contraction effectively [6, 7]. However, oxytocin desensitization may decrease effectiveness, and uterotonics also has drug contraindications and side effect such as water intoxication, nausea, vomiting, and increased blood pressure [8, 9]. Furthermore, lacking uterotonics, blood products or interventional therapy could extremely increase risk of maternal deaths in low income countries with limited resources [10]. As to PPH during vaginal delivery or after vaginal/cesarean delivery, intrauterine Bakri balloon, urgent UAE or laparotomy would be further treatment if uterotonics didn’t work, so it was important to find another conservative treatment. Given all this, the bilateral-contralateral cervix clamp was firstly introduced and applied in postpartum hemorrhage, aiming to confirm its efficacy and safety against uterotonics.

## Methods

**Fig. 1:**
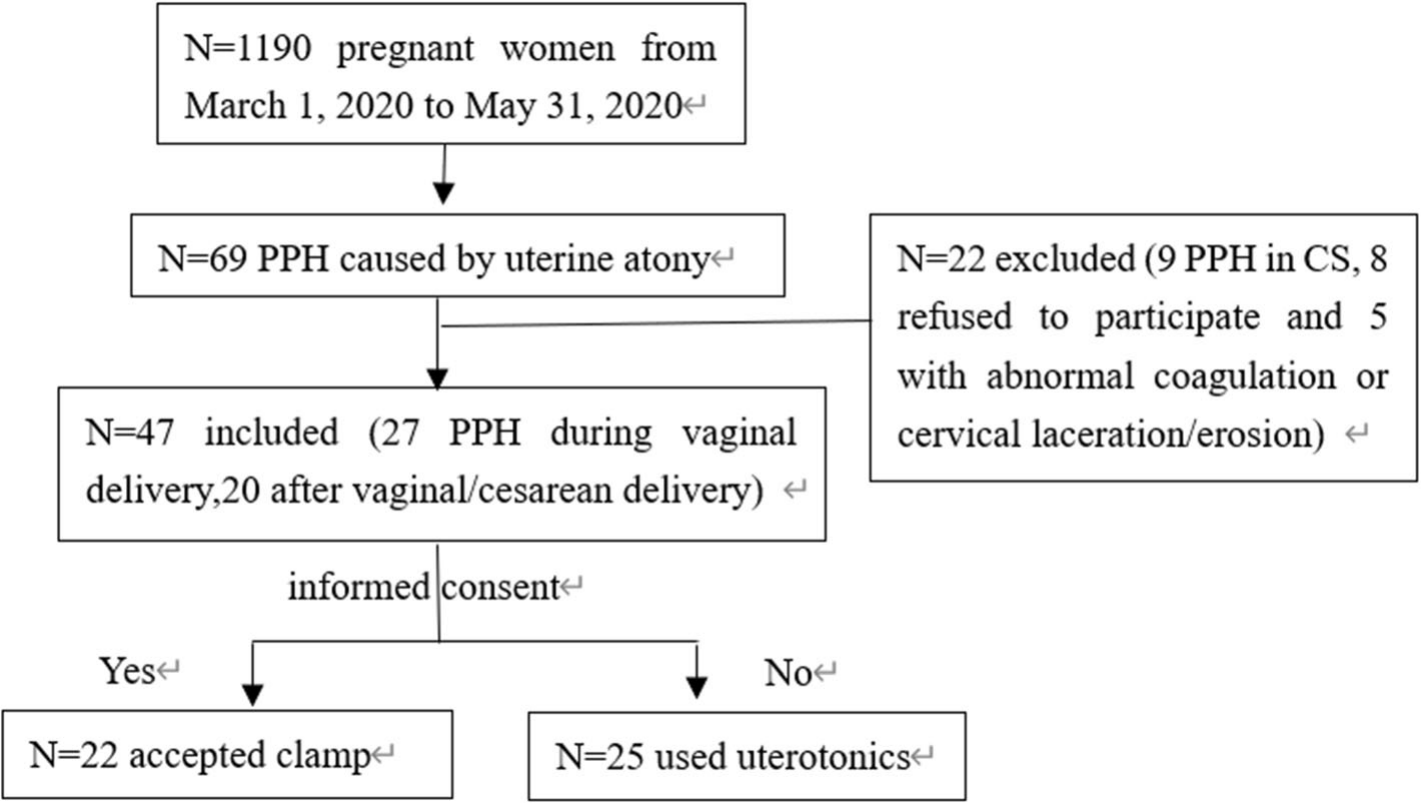
Flowchart showing the number of included and excluded subjects

The study began with approved by the Ethics Committee in West China Second University Hospital of Sichuan University (ethics number:2020068). From March 1, 2020 to May 31, 2020, there were total 69 pregnant women with PPH of 1190 delivery quantity caused by uterine atony. Among them, 22 women were excluded because 9 PPH occurred during cesarean section, 8 refused to participate and others had abnormal coagulation or severe cervical laceration/erosion. Finally 27 cases of PPH during vaginal delivery and 20 after vaginal/cesarean delivery were included. According to the informed consent principle, 22 women agreed to accept cervical clamp and 25 unwilling participants in control group used uterotonics (Fig. 1). Except routine Oxytocin, Carbetocin, Ergometrine or Hemabate was commonly used in our hospital, and further surgical procedures like intrauterine Bakri balloon, urgent UAE, laparotomy could be next treatment if needed. To increase comparability, two obstetricians finished the clamp, and three experienced midlives or obstetricians together adopted quantitative measurement to estimate blood loss, confirmed uterine atony by transvaginal uterine massage, then the nursing or epidural anesthesia was the same. The main outcomes were blood loss, uterotonic repeated usage and side effect, clamp complications, surgical procedures and hospital stay etc.

**Fig. 2:**
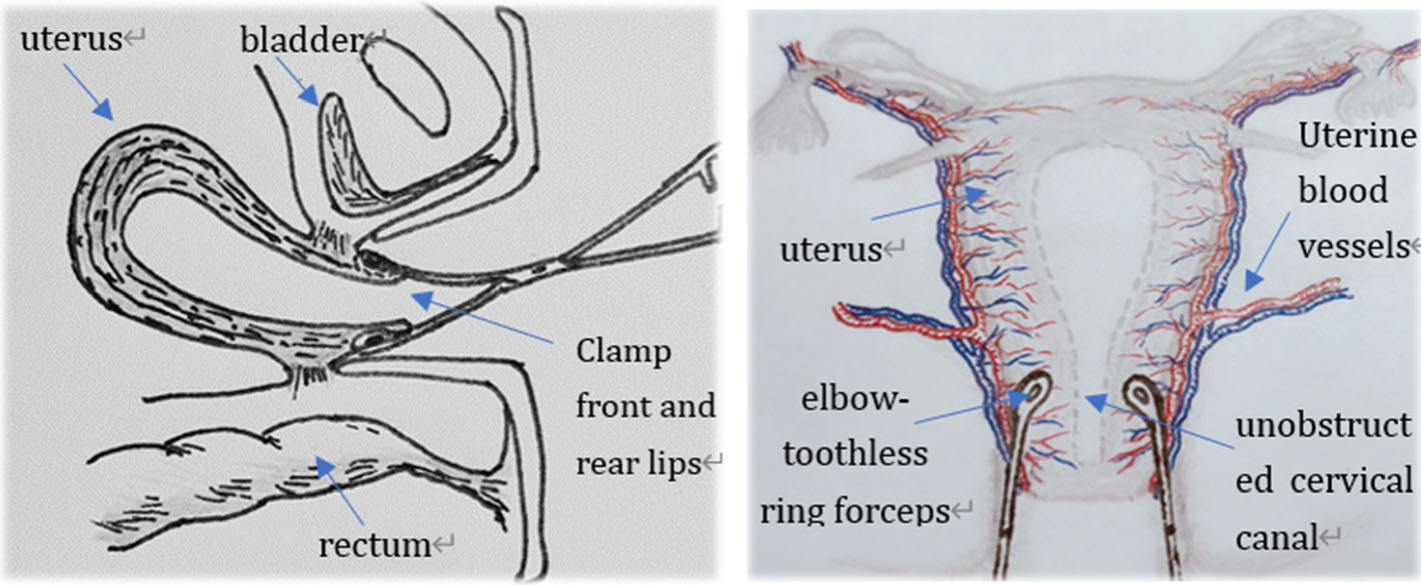
The image decapitated is our own and shows the operation points of the bilateral-contralateral cervix clamp

This bilateral-contralateral cervix clamp would be performed as first uterotonic failed or women with uterotonic contraindications. The operating steps as follows, emptying bladder after routine disinfection, vaginal speculum or tractors to expose cervix; The operator held 12 o’clock with cervical forceps, and two elbow-toothless sponge forceps to clamp front and back lip of cervix together at 3 and 9 o’clock contralaterally. The clamp depth should be 0.5 cm below the bladder reflex, and maintain 15 min with moderate closure degree. All those mentioned above contributed to blocking blood supply for lower uterine segment and stimulating endogenous oxytocin released better, preventing bladder and cervix injury as well. It was also necessary to keep cervical canal unobstructed, which was easy to observe active bleeding, timely judge hemostatic effect and if next procedure must be urgently applied or not (Fig. 2).

## Statistical analysis

Sample size calculations were performed using PASS 2011 software (NCSS, LLC, Kaysville, UT, USA). As previously described by Jiang [11], 44 women were performed BABC (Bilateral Cervix Apex Clamping) to confirm it could reduce the incidence of severe PPH and UAE. Therefore, we included 47 women in the trial, assuming a statistical difference between two groups. All statistical data were analyzed by using SPSS version 22.0 (IBM, Armonk, NY, USA), The continuous variables were presented as mean ± standard deviation (SD), whereas categorical variables were expressed as frequency, n (%), then a t-test or chi-square test might be used to compare clinical characteristics between groups. A value for p<0.05 was considered statistically significant.

## Results

Retrospective analysis of clinical data found no statistical differences on maternal characteristics between two groups, including some important factors like scarred uterus and uterotonic contraindications such as gestational hypertension, preeclampsia, heart disease, glaucoma, bronchial asthma (Table 1). Among 27 women during vaginal delivery, the intrapartum blood loss in clamp group was less than control group (656.2±72.79 g vs 811.8±86.07 g, *p* = 0.001), and even cervical clamp used in the ward, the blood loss within 30 or 30~60 min was also different between two groups (42.8±6.60 g vs 126.3±86.97 g, *p* = 0.007; 10.4±1.96 g vs 21.88±2.30 g, *P* = 0.000, respectively), but the mean hemoglobin and incidence of blood transfusion were also similar in both groups. The rate of uterotonic repeated usage (≤2 or ≥3 types/times) in clamp group was obviously lower than control group (81.8% vs 36, 18.2% vs 64%, *p* = 0.001), consequently, side effect secondary to uterotonics including water intoxication, nausea, vomiting or increased blood pressure etc. presented less in clamp group (18.2% vs 48.0%, *p* = 0.031). In addition, none of clamp complications such as failure, infection, pain, bladder or cervix injury presented, and mean hospital stay was similar in both groups (4.1±1.57d vs 3.8±1.61d, *p* = 0.535). Although intrauterine Bakri balloon or UAE had been used, there was not statistically significant between clamp and control group (4.5% vs 12%, *p* = 0.36; 4.5% vs 4.0%, *p* = 0.93, respectively), and no laparotomy in both groups (Table 2).

**Table 1:** Maternal and pregnancy characteristics of the study groups

Characteristics	Clamp group (*n* = 22)	Control group (*n* = 25)	***P*** value
**Age** (year), mean (SD)	31.0±3.62	30.9±3.65	.907
**BMI** (kg/m_2_),mean (SD)	22.5±1.66	22.2±1.85	.600
**Gravidity,** n (%)			.949
≤2	13 (59.1%)	15 (60%)	
≥3	9 (40.9%)	10 (40%)	
**Parity,** n (%)			.753
<2	14 (63.6%)	17 (68%)	
≥2	8 (36.4%)	8 (32%)	
**Pregnancy,** n (%)			.553
Single	18 (81.8%)	22 (88%)	
Twins	4 (18.2%)	3 (12%)	
**Delivery method,** n (%)			.706
Cesarean delivery	10 (45.5%)	10 (40%)	
Vaginal delivery	12 (54.5%)	15 (60%)	
**Scarred uterus**, n (%)	8 (36.4%)	6 (24%)	.355
**ART** n (%)	7 (31.8%)	5 (20%)	.354
**PROM** n (%)	7 (31.8%)	8 (32%)	.989
**ICP** n (%)	2 (9.1%)	3 (12%)	.746
**Uterotonic contraindications**, n (%)	12 (54.5%)	10 (40%)	.319
**Abnormal placenta**, n (%)			.749
Placental adhesion	6 (27.3%)	8 (32%)	
Low placenta	2 (9.1%)	1 (4%)	
Placenta previa	2 (9.1%)	2 (8%)	
**Surgical measures in groups,** n (%)			
Repair of lower uterus	6 (27.3%)	6 (24%)	.651
Uterine binding	6 (27.3%)	7 (28%)	.956
Uterine cavity packing	8 (37.4%)	3 (12%)	.105
Uterine suture	1 (4.5%)	6 (24%)	.049^a^
Ligation of uterine artery	0	3 (12%)	.046^a^
**Newborn weight** (g), mean (SD)	2832±1067	3060±1039	.706

*Abbreviations*: *BMI* Body mass index, *ART* Assisted reproductive technology, *PROM* Premature rupture of membranes, *ICP* Intrahepatic cholestasis of pregnancy, *SD* Standard deviation, *CS* Caesarean section; Uterotonic contraindications such as Gestational hypertension, Preeclampsia, Heart disease, Glaucoma, Bronchial asthma etc. ^a^ Denotes significant values if *p* < 0.05

**Table 2:** Comparison of treatment between clamp and control groups

	Clamp group (***n*** = 22)	Control group (***n*** = 25)	***P*** value
**Bleeding in labor (*n*** **= 27)**	**(*n*** **= 12)**	**(*n*** **= 15)**	
Intrapartum blood loss (g), mean (SD)	656.2±72.79	811.8±86.07	.001^a^
**Bleeding in the ward (*n*** **= 20)**	**(*n*** **= 10)**	**(*n*** **= 10)**	
Blood loss in 30 min (g), mean (SD)	42.8±6.60	126.3±86.97	.007^a^
Blood loss in 30~60 min (g), mean (SD)	10.4±1.96	21.88±2.30	.000^a^
**Hemoglobin** (g/L),mean (SD)			
Preoperative Hb	119±4.10	121.4±4.19	.058
Postoperative Hb	96.9±8.33	96.1±7.39	.718
**Blood transfusion**, n(%)	2 (9.1%)	3 (12%)	.746
**Repeated usage of uterotonic,** n(%)			.001^a^
≤2 (type/time)	18 (81.8%)	9 (36%)	
≥3 (type/time)	4 (18.2%)	16 (64%)	
**Uterotonic side effect,** n(%)	4 (18.2%)	12 (48%)	.031^a^
**Clamp complications,** n(%)	0	–	
**Surgical procedures,** n(%)			
Intrauterine Bakri balloon	1 (4.5%)	3 (12%)	.36^a^
Urgent UAE	1 (4.5%)	1 (4.0%)	.93^a^
Laparotomy	0	0	–
**Hospital stay** (days), mean (SD)	4.1±1.57	3.8±1.61	.535

*Abbreviations*: *Hb* Hemoglobin, *UAE* Uterine artery embolization, *SD* Standard deviation; ^a^ Denotes significant values if *p* < 0.05

## Discussion

Uterine atony accounted for 70~80% of etiology in postpartum hemorrhage, and the incidence of uterine atony was continuously increasing [12, 13]. In 2019, the rate of postpartum hemorrhage in West China Second Hospital of Sichuan University was about 6.0%, and uterine atony was the leading cause. Currently, active management of the third stage of labor was recommended as a prevention to reduce postpartum hemorrhage such as oxytocin administration, uterine massage and umbilical cord traction [14–16]. Except for Oxytocin, almost 3~25% of cases in PPH required another uterotonic including ergonovine, carbetocin, 15-methyl prostaglandin F2-a or misoprostol [17]. But some problems could not be overlooked, gestational hypertension, preeclampsia, heart disease, glaucoma, asthma and drug hypersensitivity were uterotonic contraindications, and drug life-time limits its repeated usage in a short time, uterotonics also brought side effect like nausea, vomiting, diarrhea, headache and increased blood pressure [8], a systematic review in 2015 even found no satisfactory evidence could suggest what kind of uterotonic was the most effective for uterine atony [18]. Two previous studies mentioned so-called clamp for fixing intrauterine balloon, or recommending Bilateral Cervix Apex Clamping (BCAC) procedure as a noninvasive therapy for severe postpartum hemorrhage [11, 19], both operations were completely different from bilateral-contralateral cervix clamp we proposed. The present study found this new technique could effectively reduce blood loss at third stage of labor or after vaginal/caesarean birth, and it was much safer than uterotonics. However, the bilateral-contralateral cervix clamp could not be recommended during caesarean section, because it was not a more rapid, immediate choice in comparison with surgical measures.

Maternal deaths caused by postpartum hemorrhage still varied in regions with different medical levels, compared with 31% of maternal deaths in Asia, 21% in Latin America, 34% in Africa were caused by postpartum hemorrhage, however, only 13% in developed countries such as the United States and 18% in France [20, 21]. If uterotonics failed, intrauterine Bakri balloon and invasive management such as uterine artery intervention or laparotomy may be recommended as second-line therapy to treat refractory hemorrhage [22, 23]. It was reported that 86% of women who had balloon tamponade did not require further procedures, and the success rate of UAE was greater than 80%, but UAE required rapid access to computed tomography and interventional radiologist, not available to all hospitals [24, 25]. Lack of uterotonics, Bakri balloon and UAE, maternal women in low income areas would be in high risk of hysterectomy and death. As for bilateral-contralateral cervix clamp, only two sponge forceps required for hospitals, also no extra charge for patients, and trained obstetrician/midwife could accomplish in 5 min, so this new technique could be another uterus-preserving management at basic-level hospital. Because no extra nursing or hospital stay required, there was also no increased burden for patient.

Even the present study was a retrospective cohort study, several ways increased its strengths. First of all, both inclusion and exclusion criteria were strictly made, three experienced midwives or obstetricians together improve accuracy of blood loss, and quantitative analysis helped directly confirm its efficacy. Then comparison of side effect, complications and cost indirectly explained its advantage. The limitations of this study included a small sample size, lacking randomization and long-term follow up. Most of all, present study couldn’t prove it work in severe PPH (≥1000 ml), or reducing incidence of intrauterine Bakri balloon, UAE or laparotomy. The future study will make perspective randomized controlled trial (RCT) and increase samples.

## Conclusions

The bilateral-contralateral cervix clamp was simple, effective, safe, low-cost, which could be a complementary treatment for PPH secondary to uterine atony at third stage of labor or after delivery, especially appropriate for pregnant women with uterotonic contraindications and hospital in poor conditions.

## Data Availability

The data of study are not publicly available due to ethical and legal restrictions. However, upon request, data may be available from Institutional Review Board of West China Second Hospital of Sichuan University.

## Abbreviations

PPH: Postpartum hemorrhage
UAE: Uterine artery embolization
CS: Caesarean section
BMI: Body mass index
ART: Assisted reproductive technology
PROM: Premature rupture of membranes
ICP: Intrahepatic cholestasis of pregnancy
SD: Standard deviation
